# Perceived Needs of Individuals with Frailty and Their Caregivers During the Transition from Hospital to Home: Protocol of a Qualitative Systematic Review and Evidence Synthesis

**DOI:** 10.3390/nursrep16020064

**Published:** 2026-02-13

**Authors:** Johanna Castro, Janeth Solís-de-Ovando, Enzo Sanfurgo, Catalina Riffo, Lucía Catalán

**Affiliations:** 1 Faculty of Healthcare Sciences, Universidad San Sebastián, Santiago 7510157, Chile; johanna.castro@uss.cl (J.C.); jsolisc@docente.uss.cl (J.S.-d.-O.); esanfurgoe@correo.uss.cl (E.S.); criffor2@correo.uss.cl (C.R.); 2 Millennium Institute for Care Research (MICARE), Santiago 8370146, Chile; 3 Doctoral Programme in Nursing Science, Faculty of Nursing, Universidad Andrés Bello, Santiago 8370146, Chile

**Keywords:** caregivers, frailty, transitional care, needs, perception, inpatient, hospitals

## Abstract

**Background:** Frailty markedly increases risk of unplanned readmission, 30-day mortality, and discontinuity of care during the transition from hospital to home. Although this transition represents a critical period for patient safety and recovery, the specific needs perceived by individuals with frailty and their caregivers at the time of discharge remain insufficiently understood. **Aim:** The aim of this study was to explore and synthesize qualitative evidence of perceived needs by individuals with frailty and their caregivers during the transition from hospital to home. **Methods:** A qualitative systematic review will be conducted following the Joanna Briggs Institute (JBI) methodological guidance. Systematic searches will be performed in PubMed, CINAHL, Web of Science (WoS), Biblioteca Virtual de Salud (BVS), Scopus, and Google Scholar. All primary qualitative and mixed-methods studies with a qualitative component, published in any language and without date restrictions, will be eligible. Methodological quality will be appraised using the JBI Critical Appraisal Checklist for Qualitative Research. A meta-aggregative approach will be applied to extract and synthesize findings using JBI SUMARI software. Confidence in the synthesized findings will be assessed using the ConQual approach. Expected results: The results will describe the perceived needs of this population and their caregivers and will support the development of practical recommendations for transitional care. **Conclusions:** The findings are expected to inform person-centered transitional care practices and support the development of clinical strategies to improve continuity of care.

## 1. Introduction

Population aging is reshaping clinical practice and the priorities of health systems, demanding a shift from disease-centered approaches toward functional capacity, continuity of care, and integrated models focused on older people [1,2]. Initiatives such as Age-Friendly Health Systems (4Ms: What Matters, Medication, Mentation, Mobility) and the World Health Organization (WHO) frameworks for “healthy ageing” reflect this paradigm shift and its growing adoption in both hospital and outpatient settings, aiming to address multimorbidity, polypharmacy, and the cumulative risks associated with this stage of life [3,4,5].

In this context, frailty stands out as a prevalent geriatric syndrome affecting a growing proportion of older adults worldwide, highlighting its magnitude and relevance as a public health issue [6]. Based on data from 62 countries, the prevalence of frailty has been estimated at 12% using the physical frailty model and at 24% using the deficit accumulation model among individuals aged 50 years and older, reaching up to 30% of the hospitalized population [7]. It is characterized by a decline in physiological reserves and increased vulnerability to health complications, which translates into higher morbidity, mortality, and disability [8]. As a result, this population faces a greater risk of hospitalization [9], and those hospitalized with frailty often experience functional decline, longer stays, and adverse events more frequently than individuals without frailty [10,11,12].

Hospitalized individuals with frailty have approximately twice the risk of 30-day unplanned rehospitalization and twice the risk of 30-day mortality compared with non-frail individuals [13,14]. Therefore, the transition from hospital to home is a particularly critical period, during which ensuring adequate discharge preparation and effective intersectoral coordination are essential to maintain continuity of care and reduce the risk of complications [15,16]. During this transition, this population faces multiple simultaneous challenges, such as adjustments to daily routines, medication management, mobility limitations, home adaptation, and emotional coping, that may further intensify pre-existing vulnerability. Recent literature documents facilitators for adequate transition, such as clear communication, staged preparation, post-discharge follow-up, and coordination with primary care and social services, yet findings remain fragmented across disciplines and contexts [16,17]. Therefore, the timely implementation of targeted interventions during this period is essential to mitigating the risks of rehospitalization and other adverse outcomes and requires careful consideration of the perceptions, experiences, and needs of these patients and their caregivers.

Although there are reviews of transitional interventions and qualitative studies on specific experiences [16,18,19], to date, no qualitative evidence synthesis has systematically examined the perceived needs of individuals with frailty and their caregivers specifically during the hospital-to-home transition. This meta-synthesis addresses this gap by integrating the perspectives of individuals with frailty and their caregivers, with the aim of translating experiential qualitative data into actionable domains for transitional nursing care, generating evidence that can guide the design and refinement of policies, clinical guidelines, and transitional care practices. By incorporating users’ experiences, transitional interventions may become more acceptable, feasible, and aligned with real-world needs, which may facilitate adherence and inform efforts to reduce complications in this vulnerable population.

The publication of this protocol adds scientific value by explicitly delineating a synthesis focus that has not been addressed systematically. Specifically, it defines frailty as a formally assessed inclusion criterion, distinguishing this review from broader syntheses focused on older adults or chronic conditions that do not explicitly characterize frailty. In addition, it restricts the phenomenon of interest to a critical, well-defined moment—the transition from hospital to home—and integrates the perspectives of individuals with frailty and their caregivers, enabling the identification of shared and divergent needs. This combination of population, context, and analytical focus constitutes the distinctive contribution of this meta-synthesis, oriented toward generating actionable domains for transitional nursing care.

### Objective

This qualitative systematic review aims to explore and synthesize qualitative evidence of perceived needs by individuals with frailty and their caregivers during the transition from hospital to home.

## 2. Materials and Methods

The review will be conducted in accordance with current methodological guidance for qualitative systematic reviews from JBI [20]. It will be reported in accordance with the Preferred Reporting Items for Systematic Reviews and Meta-Analyses Protocols (PRISMA-p) statement (Supplementary Table S1) [21]. Our review protocol has been registered with the International Prospective Register of Systematic Reviews (PROSPERO) (CRD420251145540).

### 2.1. Review Question

What are the perceived needs of individuals with frailty and their caregivers during the transition from hospital to home?

### 2.2. Search Strategy

The search strategy will be built using the PICo framework according to JBI guidelines for a qualitative systematic review, which includes Population (P), Interest phenomenon (I), and Context (Co) (Supplementary Table S2) [20]. Relevant search terms in any language will be identified in the Medical Subject Headings (MeSH) and DeCS (Acronym in Spanish for “Descriptores en Ciencias de la Salud”) databases and in key literature reviews related to the area of interest. The search will be tailored to find research specific to the views, perceptions or experiences of individuals with frailty and their caregivers regarding their needs during the transition from hospital to home. An example of the core search strategy applied across databases was as follows (All Fields): (Frailty OR “frail elderly” OR “frail older adults”) AND (Need OR “Needs assessment” OR Perception OR Requirements OR Experiences OR “Lived experiences” OR Perspective OR View) AND (“Transitional care” OR “Continuity of patient care” OR “Patient discharge” OR “hospital discharge” OR post-discharge OR “care transition”). Database-specific adaptations are provided in the supplementary material (Supplementary Table S2).

### 2.3. Eligibility Criteria

Eligible studies will explore the views, experiences, and/or perceptions of individuals with frailty and their caregivers regarding perceived needs during the transition from hospital to home. For the purposes of this review, the transition from hospital to home will be defined as the patient-centered discharge planning and discharge process from the inpatient setting to the person’s home, including preparation for discharge and the immediate post-discharge period [22]. Caregivers will be defined as individuals (family members, friends, or other unpaid/paid persons) who provide supervision, support, or assistance to a person who requires help due to illness, disability, or frailty, in the home, hospital, or institutional setting [23]. All primary qualitative or mixed-methods studies with a qualitative component published in any language will be eligible for inclusion. Primary studies published in peer-reviewed academic journals, along with theses, dissertations, and conference abstracts available from the start date of each research database, will be eligible. Editorials, personal views, case reports and documents without full text available will be excluded. For the purposes of this review, frailty will be considered a heterogeneous and multidimensional construct. Studies will be eligible only if frailty is explicitly identified as a condition through a formal assessment, regardless of the specific conceptual model applied. Details on how frailty is defined and assessed in each included study will be extracted and reported. Only studies with formally assessed frailty will be eligible; self-reported frailty, proxy indicators, and indirect inferences (e.g., based solely on age or comorbidity) will be excluded.

### 2.4. Literature Search and Study Selection

Literature search will be conducted in the National Library of Medicine (PubMed), Cumulative Index to Nursing & Allied Health (CINAHL), Web of Science (WoS), Biblioteca Virtual de Salud (BVS), Scopus and Google Scholar. For Google Scholar, the first 500 records ordered by relevance according to the platform’s default ranking will be screened to ensure transparency and reproducibility. All retrieved documents will be exported to the Rayyan platform for duplicate removal and study selection, first based on the content of titles and abstracts and then on the full text. The references of included studies will also be checked for eligibility. The entire study selection process will be conducted independently by two researchers. Any disagreements regarding eligibility will first be addressed through discussion and consensus between the two reviewers; unresolved discrepancies will be referred to a third researcher. Eligible studies will be subsequently evaluated for their methodological quality.

### 2.5. Methodological Quality

While we recognize that researchers should not automatically apply existing quantitatively informed tools to evaluate qualitative research without accounting for each study’s unique nuances, we believe it is essential to conduct some level of critical appraisal. This helps assess the trustworthiness of current evidence and supports more informed decisions about future knowledge generation. Accordingly, the JBI Critical Appraisal Checklist for Qualitative Research will be used to assess the methodological limitations of the eligible studies [24]. In accordance with JBI methodological guidance, the results of the critical appraisal will not be used as exclusion criteria but will actively inform the analytical process. Specifically, identified methodological limitations will be taken into account during the interpretation of findings, the construction of categories, and the synthesis of results and will directly inform the assessment of confidence in the synthesized findings using the ConQual approach. Studies with greater methodological limitations will therefore contribute to the synthesis with appropriate interpretive caution. Two researchers will conduct the appraisal process independently and then compare and discuss it with a third researcher. Disagreement will be resolved through discussion and consensus.

### 2.6. Data Extraction and Qualitative Synthesis

In this meta-synthesis, we will adopt the meta-aggregative approach proposed by Lockwood et al., synthesizing evidence at the level of the original authors’ interpretations and avoiding higher-order reinterpretation or theory generation [24]. According to JBI guidance, while meta-aggregation aims to preserve the original meanings of findings, it also involves a minimal level of analytical categorization to group similar findings in a coherent manner. Meta-aggregation relies on pragmatism and employs a process-oriented approach to produce findings that guide healthcare policies and practices. The recommendations from a meta-aggregative review are seen as practical, precise, detailed, and measurable because of their clear linkage to data from the included studies [25].

First, key information about each included study will be extracted using an extraction form, including author, year and type of publication, country, design, environment, phenomenon of interest, characteristics of participants, interview approaches, data analysis methods, and topics. Contextual information (e.g., country/region, care setting, and health system context as reported) will be systematically extracted and used to support interpretation during synthesis. Where the data allow, we will describe convergent and divergent needs across contexts to enhance transparency and applicability. In addition, information on how frailty is conceptualized and operationalized in each study will be systematically extracted. During data extraction, we will record whether each finding reflects the perspective of individuals with frailty, caregivers, or both. When the original study reports perspectives separately, we will extract them as separate data units. Moreover, when primary studies explicitly distinguish between informal and formal caregivers, their perspectives will be extracted and coded separately.

Qualitative findings will be extracted following the JBI recommendations through a repeated and rigorous reading of the included studies in such a way as to enable a comprehensive and contextualized representation of the findings, in line with the principles of JBI’s meta-aggregative approach. Qualitative findings will be any verbatim extract of the analytical interpretation of the results or data within the original publication. All statements, themes, or interpretations related to views, experiences and/or perceptions of individuals with frailty and their caregivers regarding perceived needs during the transition from hospital to home. Findings will be extracted with an illustration from the same study that informs the finding (e.g., a direct quotation of the participant’s voice, fieldwork observations, or other supporting data). For mixed-methods studies, only the findings related to their qualitative components will be extracted.

For each finding and its accompanying illustration (e.g., participant quote), a level of plausibility will be assigned established as follows: (I) Unequivocal (U): The findings are accompanied by a contextually rooted, detailed, rich and clearly associated illustration. (II) Credible (C): The findings are accompanied by an illustration lacking detail, richness or clear association with it. (III) Not Supported (NS). The findings are not supported by the data, or there are no data to support the finding [20]. The level of plausibility will be independently assigned to each finding and its accompanying illustration by two authors independently, and discrepancies will be resolved through discussion and consensus with a third researcher. Findings classified as III “Not Supported” will be not included in the review findings but will be reported separately for transparency.

During synthesis, studies will be grouped into analytical families according to their frailty conceptualization (e.g., physical frailty phenotype or deficit accumulation). This information will be used as an interpretive lens to explore convergences and divergences across categories and to synthesize findings, thereby supporting interpretive coherence while preserving conceptual diversity. In addition, findings from individuals with frailty and caregivers will be coded separately in the first stage and then compared and integrated to identify convergent and divergent needs; synthesized findings will report shared themes as well as any perspective-specific differences.

The software package SUMARI from JBI (https://sumari.jbi.global/, accessed on 16 October 2025) will be used to synthesize the extracted data in two parts: I. Categories will be built for findings with at least two per category grouped by conceptual similarity; II. Development of one or more synthesized findings from at least two categories. Categories will be defined/described, and synthesized findings and the explanatory statement of synthesized findings will be created by consensus among the research team members.

### 2.7. Confidence in the Findings

Synthesized findings will be graded according to ConQual by JBI to establish confidence (i.e., dependability and credibility) in the review output [26]. Credibility refers to whether the author’s interpretations align with the original data, and dependability refers to traceable variability attributed to identifiable sources. As indicated in ConQual, all findings will start with a rating of “high,” except in the case of opinion papers, which will start with a “low” level. Dependability will be established on affirmative responses to questions 2, 3, 4, 6, and 7 in the JBI Critical Appraisal Checklist: with 4 to 5 affirmative responses, dependability will be maintained (high); with 2 to 3 affirmative responses, it will be downgraded by one level (moderate); and with 0 to 1 affirmative response, it will be downgraded by two levels (low). Credibility will be determined through the level of plausibility of each synthesized finding. When there are both unequivocal and credible findings, the confidence level will be downgraded by one level (moderate). When all findings are equivocal, it will be downgraded by two levels (low). The final confidence level (High, Moderate, Low, or Very Low) will be determined by applying downgrades based on their individual dependability and credibility scores. In line with JBI guidance, the application of ConQual will not be purely mechanical. Two independent reviewers will assess credibility and dependability and will engage in reflexive dialog to reach consensus on any discrepancies. This ensures a contextualized and nuanced evaluation of confidence in the synthesized findings. This procedure is explained in detail in Munn et al. (2014) [26].

In this review, each synthesized finding will be presented and discussed, along with the type of research informing it, its dependability and credibility scores, and its overall ConQual score.

## 3. Expected Results

The results will include synthesized categories and findings that will describe the perceived needs of individuals with frailty and their caregivers during the transition from hospital to home. Following the JBI meta-aggregative approach, these findings will reflect patterns of meaning and lived experiences reported in the primary studies, without attributing causal effects. The structured presentation of the results will enable their future translation into practical recommendations for clinical care and service organization in transitional care.

## 4. Discussion

The experiences and needs of individuals with frailty and their caregivers during the transition from hospital to home have been described in the literature as a critical aspect of care quality, with potential implications for safety, continuity of care, and recovery. The literature shows that insufficient discharge preparation and weak intersectoral coordination are associated with increased risks of readmissions, adverse events, and caregiver burden [15,16]. Quantitative studies have demonstrated that transitional care interventions aimed at actively engaging patients and caregivers can achieve measurable effects, such as reductions in rehospitalization or acute care use [27]. However, these outcomes-based evaluations primarily capture clinical or utilization endpoints and offer limited insight into the underlying mechanisms through which such interventions succeed or fail, particularly in the context of frailty. As a result, important user-perceived needs that may explain or hinder observed effects often remain insufficiently explored [27].

This protocol describes a qualitative systematic review designed to synthesize existing qualitative evidence on perceived needs at this critical moment, integrating the perspectives of individuals with frailty and their caregivers. Recent empirical evidence further suggests that, at hospital discharge, essential communication domains are frequently not fully addressed, including medication changes, recognition of warning signs, follow-up arrangements, and verification of patient and caregiver understanding. These gaps help explain why risks and caregiver burden may persist at home despite the presence of formal discharge strategies. Moreover, these documented challenges highlight the relevance of conducting a qualitative evidence synthesis, rather than presupposing specific unmet needs or outcomes. Exploring and synthesizing qualitatively how individuals with frailty and their caregivers experience this transition allows for an in-depth understanding of informational, emotional, and functional needs that may not be captured by “hard” outcomes such as readmission rates alone [18,28].

As a protocol, this discussion does not anticipate the specific content of the review findings but rather establishes the rationale for the synthesis and its relevance. The results of this review are expected to consist of synthesized categories and findings describing perceived needs, which may inform the development of actionable recommendations for discharge preparation, service organization, and policy, consistent with the pragmatic orientation of JBI meta-aggregation, without implying causal or effectiveness claims. Ultimately, this review seeks to contribute to a more nuanced understanding of hospital-to-home transitions for individuals with frailty and their caregivers.

### Limitations

Nevertheless, inherent limitations of the design are anticipated. The inclusion of studies in multiple languages may be affected by the availability of translations, introducing potential language bias, which may influence both the interpretation and the confidence assigned to the synthesized findings. Furthermore, methodological heterogeneity among qualitative studies—regarding contexts, designs, and theoretical frameworks—may complicate synthesis and may result in context-specific insights rather than universally generalizable conclusions. There is also a risk of underrepresentation of certain vulnerable groups, such as individuals with severe frailty or informal caregivers in rural areas, potentially limiting the applicability of some findings to particular settings or populations. As with any qualitative synthesis, findings will be limited to what primary studies report. To support a contextualized interpretation, a summary table will present key contextual and methodological characteristics of the included studies, facilitating an informed assessment of the transferability and practical relevance of the synthesized findings and resulting recommendations.

## 5. Conclusions

This review will synthesize the available evidence on the perceived needs of individuals with frailty and their caregivers during the transition from hospital to home, with the aim of contributing to a deeper understanding of these perspectives and informing the design of future interventions, such as discharge preparation strategies, that are more closely aligned with the expressed needs of this population. By incorporating these perspectives, the findings aim to generate actionable insights for policymakers, clinicians, and researchers, in line with the JBI meta-aggregative approach. This review will provide a foundation of synthesized findings that can support the development of more person-centered and contextually relevant transitional care practices.

## Data Availability

All data related to this manuscript can be accessed upon consultation with the authors.

## Supplementary Materials

The following supporting information can be downloaded at: https://www.mdpi.com/article/10.3390/nursrep16020064/s1, Table S1: PRISMA-P (Preferred Reporting Items for Systematic review and Meta-Analysis Protocols) 2015 checklist: recommended items to address in a systematic review protocol*; Table S2: Search strategies according to databases.

## Public Involvement Statement

No public involvement in any aspect of this research.

## Guidelines and Standards Statement

This manuscript will be reported in accordance with the Preferred Reporting Items for Systematic Reviews and Meta-Analyses Protocols (PRISMA-p) statement. A complete list of reporting guidelines can be accessed via the Equator Network: https://www.equator-network.org/ (Accessed on 19 December 2025).

## Abbreviations

The following abbreviations are used in this manuscript:

PubMed	National Library of Medicine
CINAHL	Cumulative Index to Nursing & Allied Health
WoS	Web of Science
BVS	Biblioteca Virtual de Salud (in Spanish)
JBI	Joanna Briggs Institute
PAHO	Pan American Health Organization
WHO	World Health Organization
PRISMA-p	Preferred Reporting Items for Systematic Review and Meta-Analysis Protocols
MeSH	Medical Subject Headings
DeCS	Descriptores en Ciencias de la Salud
PROSPERO	Prospective Register of Systematic Reviews

## References

[1] PAHO (2025). The Decade of Healthy Aging in the Americas (2021–2030)—Paho/Who|Pan American Health Organization. https://www.paho.org/en/decade-healthy-aging-americas-2021-2030.

[2] Dyer S.M., Suen J., Williams H., Inacio M.C., Harvey G., Roder D., Wesselingh S., Kellie A., Crotty M. (2022). Impact of relational continuity of primary care in aged care: A systematic review. BMC Geriatr..

[3] Fulmer T., Mate K.S., Berman A. (2018). The age-friendly health system imperative. J. Am. Geriatr. Soc..

[4] WHO (2015). World Report on Ageing and Health. https://www.who.int/publications/i/item/9789241565042.

[5] Barnett K., Mercer S.W., Norbury M., Watt G., Wyke S., Guthrie B. (2012). Epidemiology of multimorbidity and implications for health care, research, and medical education: A cross-sectional study. Lancet.

[6] O’Caoimh R., Sezgin D., O’Donovan M.R., Molloy D.W., Clegg A., Rockwood K., Liew A. (2021). Prevalence of frailty in 62 countries across the world: A systematic review and meta-analysis of population-level studies. Age Ageing.

[7] Veronese N., Custodero C., Cella A., Demurtas J., Zora S., Maggi S., Barbagallo M., Sabbà C., Ferrucci L., Pilotto A. (2021). Prevalence of multidimensional frailty and pre-frailty in older people in different settings: A systematic review and meta-analysis. Ageing Res. Rev..

[8] Martínez-Reig M., Flores Ruano T., Fernández Sánchez M., Noguerón García A., Romero Rizos L., Abizanda Soler P. (2016). Fragilidad como predictor de mortalidad, discapacidad incidente y hospitalización a largo plazo en ancianos españoles. Estudio FRADEA. Rev. Esp. Geriatr. Gerontol..

[9] Kojima G. (2016). Frailty as a predictor of hospitalisation among community-dwelling older people: A systematic review and meta-analysis. J. Epidemiol. Community Health.

[10] Faitna P., Bottle A., Klaber B., Aylin P.P. (2024). Has multimorbidity and frailty in adult hospital admissions changed over the last 15 years? A retrospective study of 107 million admissions in England. BMC Med..

[11] Madsen F., Ladelund S., Linneberg A. (2014). High levels of bed occupancy associated with increased inpatient and thirty-day hospital mortality in Denmark. Health Aff. Proj. HOPE.

[12] Tabata-Kelly M., Ruan M., Dey T., Sheu C., Kerr E., Kaafarani H., Ornstein K.A., Kelley A., Gray T.F., Salim A. (2023). Postdischarge caregiver burden among family caregivers of older trauma patients. JAMA Surg..

[13] Gregersen M., Hansen T.K., Jørgensen B.B., Damsgaard E.M. (2020). Frailty is associated with hospital readmission in geriatric patients: A prognostic study. Eur. Geriatr. Med..

[14] Hao Q., Zhou L., Dong B., Yang M., Dong B., Weil Y. (2019). The role of frailty in predicting mortality and readmission in older adults in acute care wards: A prospective study. Sci. Rep..

[15] Liebzeit D., Rutkowski R., Arbaje A.I., Fields B., Werner N.E. (2021). A scoping review of interventions for older adults transitioning from hospital to home. J. Am. Geriatr. Soc..

[16] Baxter R., Shannon R., Murray J., O’Hara J.K., Sheard L., Cracknell A., Lawton R. (2020). Delivering exceptionally safe transitions of care to older people: A qualitative study of multidisciplinary staff perspectives. BMC Health Serv. Res..

[17] Sun M., Liu L., Wang J., Zhuansun M., Xu T., Qian Y., Rosa R.D. (2023). Facilitators and inhibitors in hospital-to-home transitional care for elderly patients with chronic diseases: A meta-synthesis of qualitative studies. Front. Public Health.

[18] Couture V., Germain N., Côté É., Lavoie L., Robitaille J., Morin M., Chouinard J., Couturier Y., Légaré F., Hardy M.-S. (2024). Transitions of care for older adults discharged home from the emergency department: An inductive thematic content analysis of patient comments. BMC Geriatr..

[19] Lee J.Y., Yang Y.S., Cho E. (2022). Transitional care from hospital to home for frail older adults: A systematic review and meta-analysis. Geriatr. Nurs..

[20] Aromataris E., Lockwood C., Porritt K., Pilla B., Jordan Z. (2024). JBI Manual for Evidence Synthesis.

[21] Shamseer L., Moher D., Clarke M., Ghersi D., Liberati A., Petticrew M., Shekelle P., Stewart L.A., PRISMA-P Group (2015). Preferred reporting items for systematic review and meta-analysis protocols (PRISMA-P) 2015: Elaboration and explanation. BMJ.

[22] Coleman E.A., Boult C. (2003). Improving the Quality of Transitional Care for Persons with Complex Care Needs: Position Statement of The American Geriatrics Society Health Care Systems Committee. J. Am. Geriatr. Soc..

[23] Caregivers—MeSH—NCBI. https://www.ncbi.nlm.nih.gov/mesh/68017028.

[24] Lockwood C., Munn Z., Porritt K. (2015). Qualitative research synthesis: Methodological guidance for systematic reviewers utilizing meta-aggregation. Int. J. Evid. Based Healthc..

[25] Hannes K., Lockwood C. (2011). Pragmatism as the philosophical foundation for the Joanna Briggs meta-aggregative approach to qualitative evidence synthesis. J. Adv. Nurs..

[26] Munn Z., Porritt K., Lockwood C., Aromataris E., Pearson A. (2014). Establishing confidence in the output of qualitative research synthesis: The ConQual approach. BMC Med. Res. Methodol..

[27] Lee J.Y., Kim S., Kim G.S., Lee K.H., Kim C.O., Cho E. (2023). The effectiveness of a transitional care program for frail older adults between hospital and home: A randomized controlled trial. Geriatr. Nurs..

[28] Trivedi S.P., Corderman S., Berlinberg E., Schoenthaler A., Horwitz L.I. (2023). Assessment of Patient Education Delivered at Time of Hospital Discharge. JAMA Intern. Med..

